# Synthesis of ordered lamellar supermicroporous silica with rigid neutral and long-chain cationic composite templating route

**DOI:** 10.1371/journal.pone.0216117

**Published:** 2019-04-26

**Authors:** Shangxing Chen, Peng Wang, Guorong Fan, Shengliang Liao, Hongyan Si, Zongde Wang

**Affiliations:** Collaborative Innovation Center of Jiangxi Typical Trees Cultivation and Utilization, College of Forestry, Jiangxi Agricultural University, Nanchang, P.R. China; Massey University, NEW ZEALAND

## Abstract

Using a mixture of neutral primary amine dehydroabietylamine (DHAA) and long-chain cetyltrimethyl ammonium bromide (CTAB) as the template, ordered lamellar supermicroporous silicas were synthesized with NaOH as the base source and tetraethylorthosilicate (TEOS) as the silica source. The concentrations of DHAA, CTAB, and NaOH in the synthesis system had great effects on the structural properties of the samples. When the molar ratio of components was nTEOS:nCTAB:nDHAA:nNaOH:nH_2_O = 1:0.114:0.00457:0.5:60, the material showed a lamellar phase with the highest ordering degree. By adding only a trace amount of DHAA into the synthesis system, the structure of the samples could be transformed from cubic phase to lamellar phase, since the added DHAA solubilized in CTAB micelles to change the effective surfactant ion pair packing parameter. The dosage of CTAB should be moderate; too high or too low will decay the ordering degree of the lamellar structure.A much higher concentration of NaOH resulted in an ethanol-rich solvent in which the DHAA did not solubilize in the micelles of CTAB, but adsorbed at the hydrophilic headgroup–solvent interface. Accordingly, a structural transformation from lamellar phase to hexagonal phase occurred.

## Introduction

According to the International Union of Pure and Applied Chemistry (IUPAC) classification, materials with pore size less than 2 nm can be denoted as microporous materials, while materials with pore size in the range of 2–50 nm are mesoporous materials[1]. Usually, the pore size of ordinary microporous zeolites does not break the boundary of 1.2 nm. Meanwhile, it is also very hard to fabricate well-ordered mesoporous materials with pore size less than 2.0 nm through the ordinary templating route[2]. Actually, materials with pore size of 1.2~2.0 nm, which can be denoted as supermicroporous materials, are important due to their application potential in the field of shape-selective catalysis as well as adsorption and separation of compounds with specific molecular sizes[3, 4]. Therefore, there is an urgent demand for the preparation of ordered supermicroporous materials to fill the gap between microporous zeolites and mesoporous molecular sieves. However, because of the lack of suitable templating agents, synthesizingordered supermicroporous materials is still a scientific challenge[5].

In order to obtain supermicroporous structures, short-chain templating agents are usually required. Unfortunately, it is difficult to form well-ordered supermicroporous structures owing to their poor self-assembly ability[6–8]. In order to solve this problem, a variety of methods have been adopted, such as pore-opening size tailoring[9], high-temperature treatment with stabilizer[10], composite surfactant templating[11–13], and elaborately designed surfactant templating methods[14–16]. Among them, the composite surfactant templating method is more acceptable because of the simple synthesis process and commercially available reagents. Lin et al.[11] reported the synthesis of ordered supermicroporous aluminosilicate by using short-chain decyltrimethyl ammonium bromide and butanol as the mixing templating agents. The introduction of butanol significantly improved the regularity of the materials, because it functioned as a co-surfactant in the palisadelayer to decrease the interfacial curvature and elongate the micelles of decyltrimethyl ammonium bromide. Wang et al.[12] reported the preparation of supermicroporous silica with a short-chain cationic–anionic templating agent. Under electrostatic attraction, the addition of anionic surfactant into the cationic system resulted in a smaller effective headgroup area by reducing the distance between the surfactant heads. Therefore, well-ordered hexagonal supermicroporous silica was obtained. Dodecyltriethyl ammonium bromide was used as template agent by Fu et al.[13] to fabricateordered cubic supermicroporous aluminosilicates with bifunctionalassistance of succinic acid and malonic acid(weak acid media and co-surfactant). Such polycarboxylic acids stayed at the outer layer of the micelle to increase the effective headgroup area, which was conducive to the formation of the cubic mesostructure. Obviously, the presence of the co-surfactants hada great effect on the packing behavior of the mixing templating micelles to obtain different kinds of pore structures. Until now, no reports onthe synthesis of lamellar supermicroporous silica, especially the rigid neutral and long-chain cationic kind,bythe neutral-cationic composite templating route have appeared in the literature.

Previously, our group successfully synthesized ordered hexagonal silica with rosin-based quaternary ammonium salt made from dehydroabietylamine (DHAA; the molecularstructure is shown in S1 File)[17]. In this paper, by mixing cetyltrimethyl ammonium bromide (CTAB) and DHAA as the templating agents, ordered lamellar supermicroporous silicas were synthesized. The effects of the molar ratios of CTAB, DHAA, tetraethylorthosilicate (TEOS), and NaOH were investigated, and the probable phase transformation mechanism of the materials was discussed.

## Experimental

### Materials

CTAB (AR), NaOH (AR), and TEOS (AR) were provided by Sinopharm Chemical Reagent Co. Ltd. Dehydroabietylamine (~98 wt%) was purchased from Hangzhou Wanjing New Materials Co. Ltd.

### Synthesis

Certain amounts of CTAB, DHAA, and NaOH were dissolved in distilled water under vigorous stirring at 308 K. TEOS was slowly added into thissolution, and then a white precipitate was obtained. The molar ratio of the resulting mixture was nTEOS:nCTAB:nDHAA:nNaOH:nH_2_O = 1:0.026~0.1425:0~0.01428:0.417~0.65:60. After continuous stirring for 5 h, the mixture was transferred to a Teflon-lined steel autoclave and subjected to hydrothermal treatment in air-dry oven at 373 K for 72 h. The resulting product was filtered out and washed with distilled water and ethanol several times. After drying at 333 K overnight, the organic species were removed by extraction in ethanol–HCl for 8 h or calcination in air at 823 K for 4 h.

### Characterization

X-ray diffraction (XRD) characterizations of the samples were recorded by a D8 Focus diffractometer (Bucker AXS Inc., Germany) with Cu Ka radiation (k = 0.154 nm). The operating target voltage was 40 kV and the current was 40 mA. The sample was scanned for 2*θ*, ranging from 0.5° to 10.0° for small-angle. Transmission electron microscopy (TEM) images were obtained with a JEM-2100 instrument (JEOL Ltd., Japan) operated at an accelerating voltage of 200 kV. The samples were ultrasonically dispersed in ethanol and then dropped onto carbon-coated copper grids prior to measurement.Nitrogen sorption isotherms were measured on a Micromeritics ASAP2020 system (Micromeritics Instrument Corp., USA).The surface areas were calculatedusing the BET method, and the poredistributions were plotted using the DFT model.

## Results and discussion

### DHAA concentration

**Fig 1:**
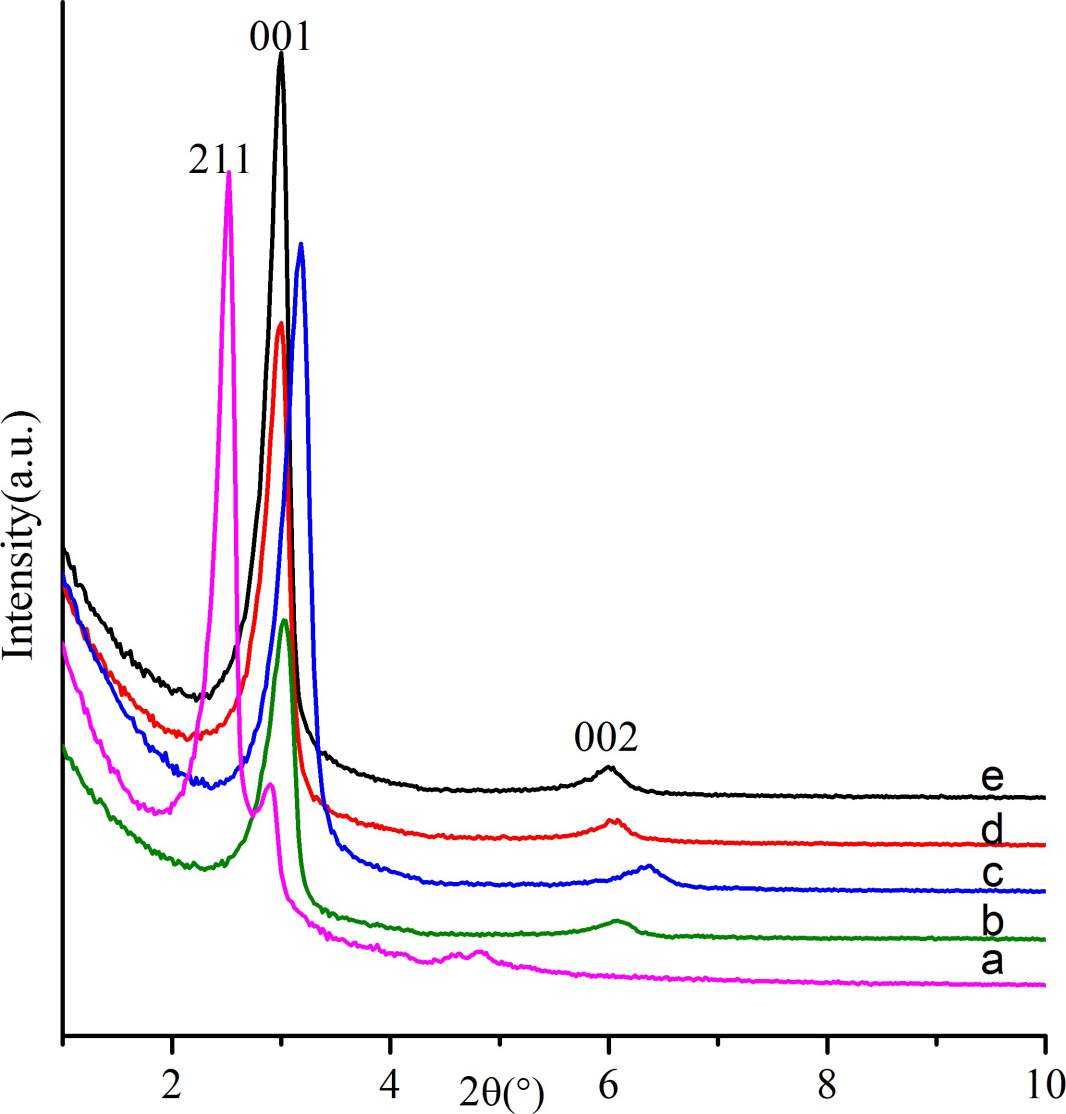
Small-angle XRD patterns of as-synthesized silicas prepared with the following conditions: nTEOS:nCTAB:nDHAA:nNaOH:nH_2_O = 1:0.114:x:0.5:60: (**a**) x = 0, (**b**) x = 0.00286, (**c**) x = 0.00457, (**d**) x = 0.00714, (**e**) x = 0.01428.

Fig 1 shows the small-angle XRD patterns of samples synthesized with different DHAA concentrations. The sample without the addition of DHAA (Fig 1A) reveals an identical cubic MCM-48 pattern with a strong diffraction peak at 2*θ* = 2.52° (d_211_ = 3.50 nm), a weak shoulder diffraction peak at 2*θ* = 2.90° (d_220_ = 3.04 nm), and some slight diffraction peaks at 2*θ* = 3.85°~5°. As the DHAA was added to the synthesis system, the sample (Fig 1B) showed two diffraction peaks at 2*θ* = 3.04° (d_001_ = 2.90 nm) and 2*θ* = 6.08° (d_002_ = 1.45 nm), which could be indexed on a lamellar phase. In contrast with the conventional MCM-50[18], the position of the (001) diffraction peak for our sample shows at a much higher angle, which is similar to the results obtained by Zhou et al.[19], indicating that an ordered lamellar supermicroporous material was prepared. When the molar ratio of DHAA increased to 0.00457, the diffraction peaks became stronger and sharper, suggesting increasedregularity of the supermicroporous structure. With the further increase of DHAA concentration, the samples (Fig 1C–1E) still possessed well-ordered supermicroporous structure, but no significant improvements could be observed in the regularity of the materials. It can be seen from the above results that even a trace amount of DHAA can lead to a structural transformation from cubic phase to lamellar phase. Such a phenomenon may be caused by the solubilization of DHAA in CTAB micelles, which changed the effective surfactant ion pair packing parameter, g = V/a_0_l_c_ (V, hydrophobic chain volume; a_0_, effective area of polar head;l_c_, length of hydrophobic chain)[20]. First of all, the primary amine groups of DHAA will insert into the voids between the headgroups of CTAB through electrostatic interaction, which will decrease the distancebetween the headgroups. Therefore, the a_0_ value decreased accordingly. Second, DHAA has a hydrophobic three-ringphenanthrene-like skeleton (composed of a benzene ring and two saturated six-membered rings), which is rigid and haslarge volume. Such hydrophobic groups will stretch into the hydrophobic centers of the CTAB micelles to increase the V value. Meanwhile, with the *π* electrons from the benzene ring, the hydrophobic group of DHAA can interact with the alkyl chain of CTAB through the powerful van der Waals force. Because DHAA possesses a short molecular diameter (less than 2 nm), the alkyl chain of CTAB will shrink and bind onto the hydrophobic group of DHAA in water to form a smaller hydrophobic core. As a result, the length of the hydrophobic chain (l_c_) was reduced, and the pore size of the materials decreased. Finally, the g value increased to about 1, and well-ordered lamellar silica with smaller pore size was obtained.

### CTAB concentration

**Fig 2:**
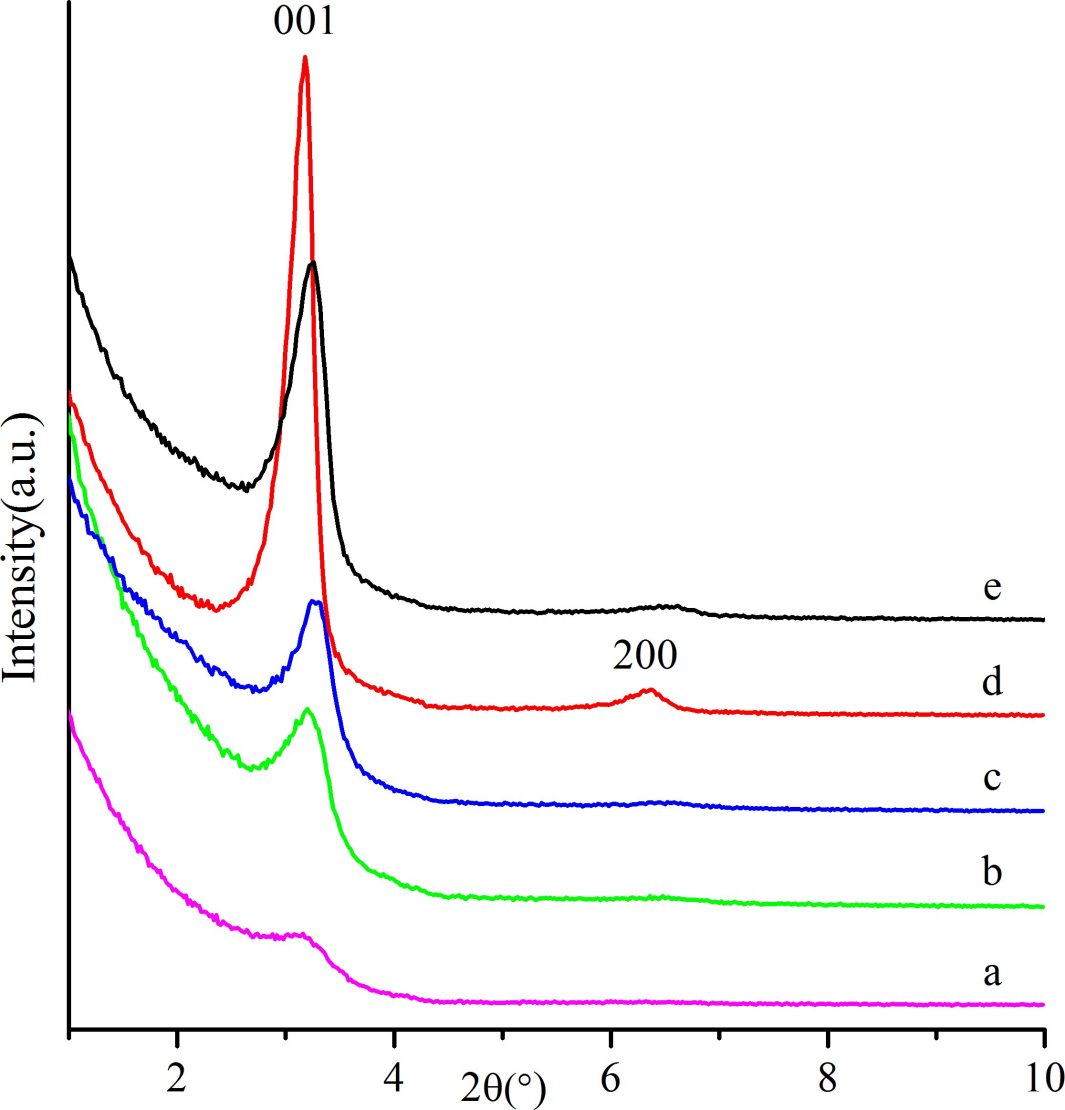
Small-angle XRD patterns of as-synthesized silicas prepared with the following conditions: nTEOS:nCTAB:nDHAA:nNaOH:nH_2_O = 1:x:0.00457:0.5:60: (**a**) x = 0.026, (**b**) x = 0.057, (**c**) x = 0.0741, (**d**) x = 0.114, (**e**) x = 0.1425.

Small-angle XRD patterns of samples prepared with various CTAB dosages are shown in Fig 2. As the amount of added CTAB was smaller, the sample (Fig 2A) showed only a weak and broad peak at 2*θ* = 3.16° (d_001_ = 2.97 nm), indicating that the regularity of the pore structure was poor. Obviously, the formation of an ordered pore structure requires a sufficient amount of CTAB in the reaction system. When the content of CTAB increased from 0.026 to 0.0741, the peaks of the samples (Fig 2B and 2C) gradually strengthened. When the dosage of CTAB was 0.114, the sample (Fig 2D) presented two peaks at 2*θ* = 3.18° (d_001_ = 2.78 nm) and 2*θ* = 6.38° (d_002_ = 1.38 nm), suggesting thata highly ordered supermicroporous silica was obtained. However, the further addition of CTAB led to decreaseddiffraction peaks (Fig 2E). The reason for this result may be that the excessive CTAB occupied the space of silicate species, which restricted the interaction between the micelles of the templating agent and silicate species. Therefore, the regularity of the pore structure wasdecreased.

### NaOH concentration

**Fig 3:**
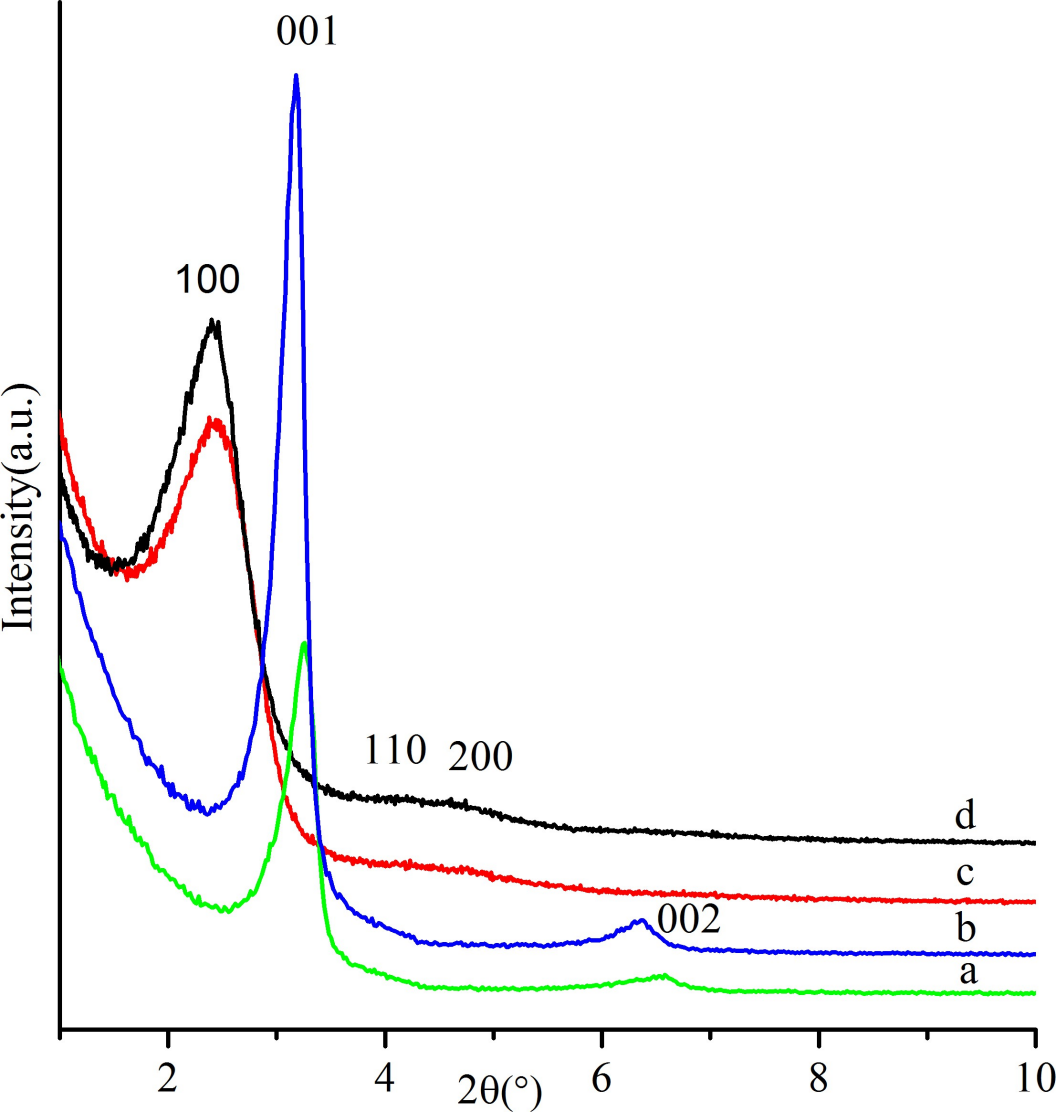
Small-angle XRD patterns of as-synthesized silicas prepared with the following conditions: nTEOS:nCTAB:nDHAA:nNaOH:nH_2_O = 1:0.114:0.00457:x:60: (**a**) x = 0.417, (**b**) x = 0.5, (**c**) x = 0.6, (**d**) x = 0.65.

Fig 3 illustrates the small-angle XRD patterns of samples prepared with different NaOH concentrations. When the NaOH content was 0.417, the XRD pattern (Fig 3A) for the prepared silica showed two peaks at 2*θ* = 3.26° (d_001_ = 2.71) and 2*θ* = 6.58° (d_002_ = 1.34), indicating the formation of a lamellar supermicroporous structure. As the NaOH content increased to 0.5, two stronger peaks at 2*θ* = 3.18° (d_001_ = 2.78 nm) and 2*θ* = 6.38°(d_002_ = 1.38 nm) can be observed in the pattern in Fig 3B, suggesting that a suitable increase of NaOH is beneficial to the enhancement of regularity. As shown in Fig 3C and 3D, with the further increase of NaOH content to 0.56 and 0.65, a broad reflection peak at 2*θ* = 2.41° (d_100_ = 3.66 nm)and two weak peaks at 2*θ* = 4°~5°could be detected for both samples, which can be indexed as 100, 110, and 200 crystal planes of the hexagonal phase. Thus, as a result of increasing pH value, the structuraltransformation from lamellar phase to hexagonal phase occurred. As the dosage of NaOH increased, the hydrolysis rate of TEOS increased accordingly, which led to a higher content of ethanol in the synthesis system. In such an ethanol-rich solvent, the solubility of DHAA will be increased. Therefore, the DHAA will not solubilize in CTAB micelles, but adsorb at the hydrophilic headgroup–solvent interface, leading to an increase in the effective area of polar head (a_0_), elongation of the length of hydrophobic chain (l_c_), and reduction of the hydrophobic chain volume (V). Therefore, the g value decreased to 1/2 accordingly, and an MCM-41–like hexagonal silica was obtained. However, because of the existence of DHAA, the interaction between the silicate species and the headgroupsof the templating agentswas hindered, so that the ordering degree of the obtained materials was poor.

### Templating agent removal

**Fig 4:**
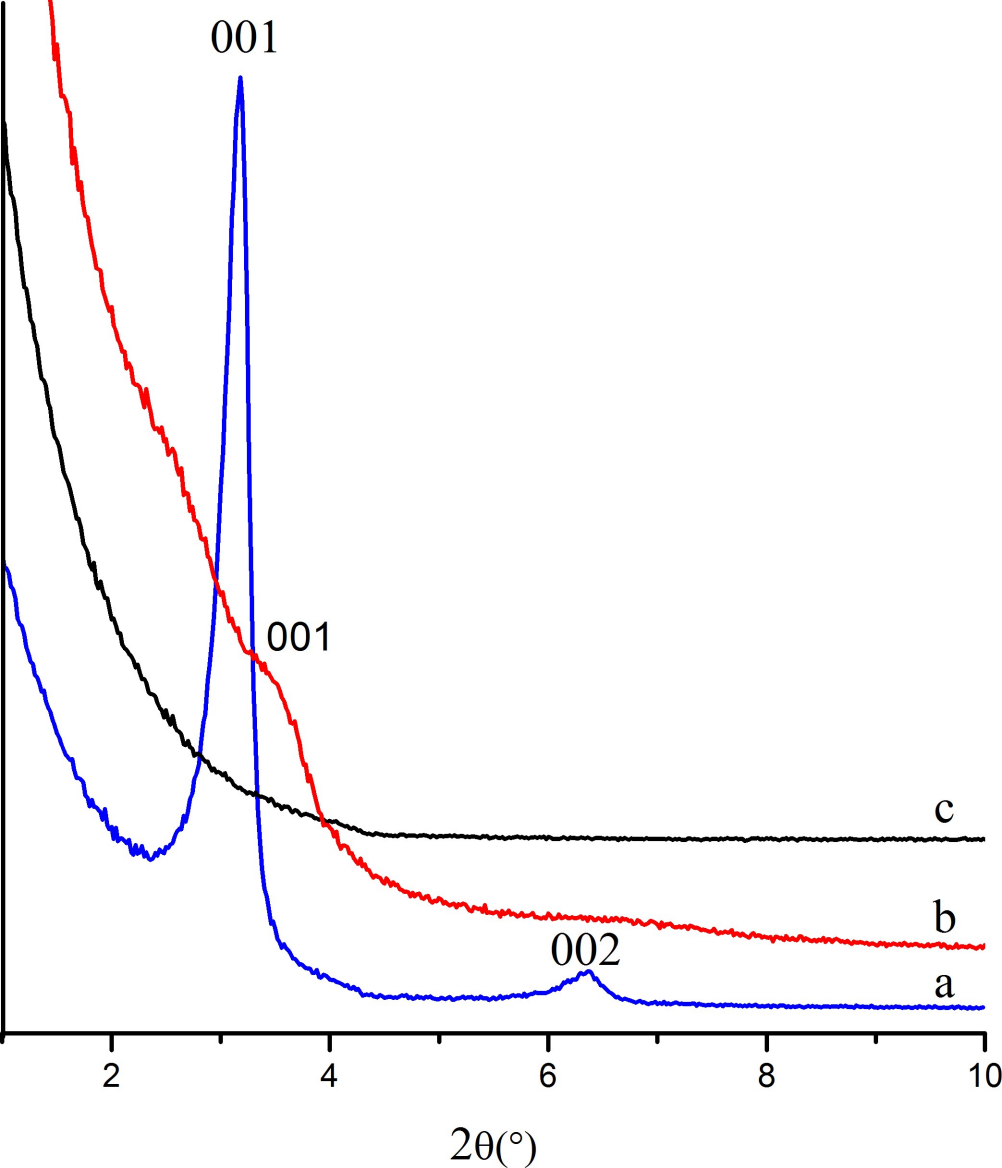
Small-angle XRD patterns of (**a**) as-synthesized (**b**) ethanol–HClextracted,and (**c**)calcinedsilicas prepared with the following condition: nTEOS:nCTAB:nDHAA:nNaOH:nH_2_O = 1:0.114:0.00457:0.5:60.

Fig 4 shows the small-angle XRD patterns of as-synthesizedethanol-HCl extractedand calcined samples prepared with a mole ratio of nTEOS:nCTAB:nDHAA:nNaOH:nH_2_O = 1:0.114:0.00457:0.5:60. After extraction in refluxed ethanol–HCl for 8 h, sample b (Fig 4B) showed only a broad diffraction peak at 2*θ* = 3.40° (d_001_ = 2.60 nm), suggesting that the ordered pore structure of the sample was partially destroyed by the extraction. After calcination at 823 K for 4 h, no diffraction peaks could be observed (Fig 4C), indicating that the lamellar pore structurefully collapsed. Just like conventional lamellar mesoporous materials, such as MCM-50[18], our samples also could not retain ordered pore structure after removal of the templating agent.

### TEM characterization

**Fig 5:**
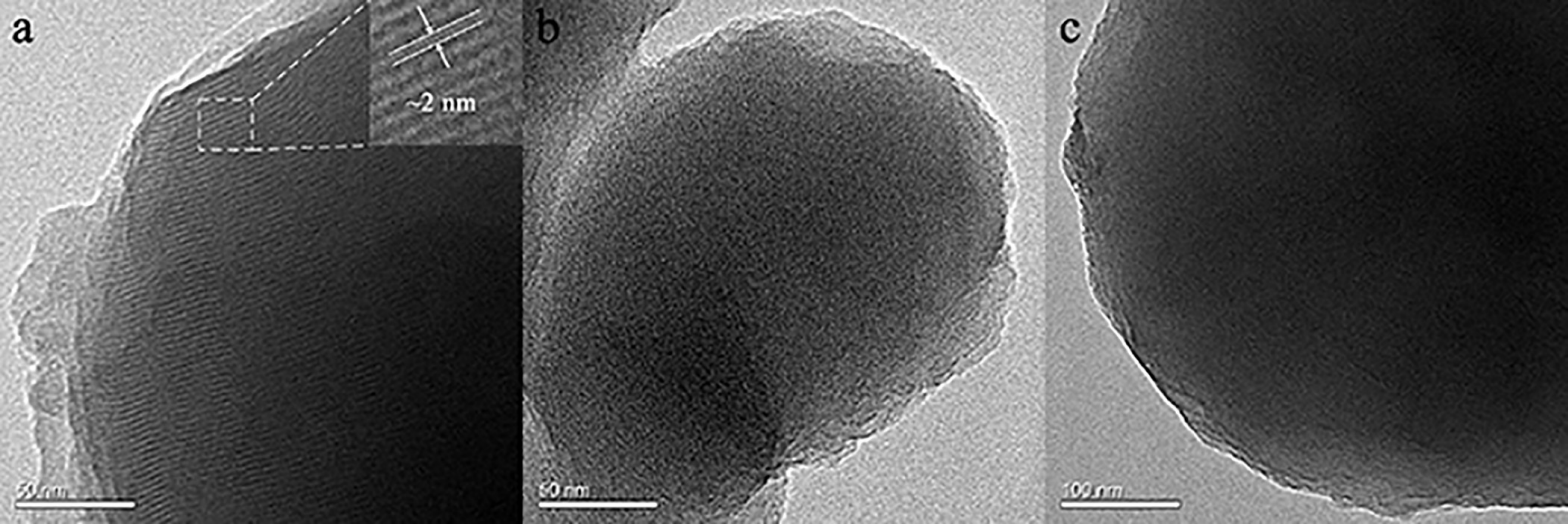
TEM images of (**a**)as-synthesized, (**b**)ethanol–HClextracted and (**c**) calcinedsilicas with the following condition: nTEOS:nCTAB:nDHAA:nNaOH:nH_2_O = 1:0.114:0.00457:0.5:60.

Fig 5 shows the TEM images of as-synthesized, ethanol–HClextracted, and calcined samplesprepared with the molar ratio of nTEOS:nCTAB:nDHAA:nNaOH:nH_2_O = 1:0.114:0.00457:0.5:60. It can be seen that the channels of the as-synthesized sample(Fig 5A) are plotted in parallel with a long-range order, verifying the lamellar nature of the pore structure. The pore size was about 2 nm, in the supermicroporous range.However, after extracting in ethanol–HCl, the pore structure of the sample was partially destroyed (Fig 5B), and after calcination at 823 K for 4 h, the pore channels collapsed completely (Fig 5C).Such resultsare consistent with the XRD characterization discussed above.

### N_2_ sorption characterization

**Fig 6:**
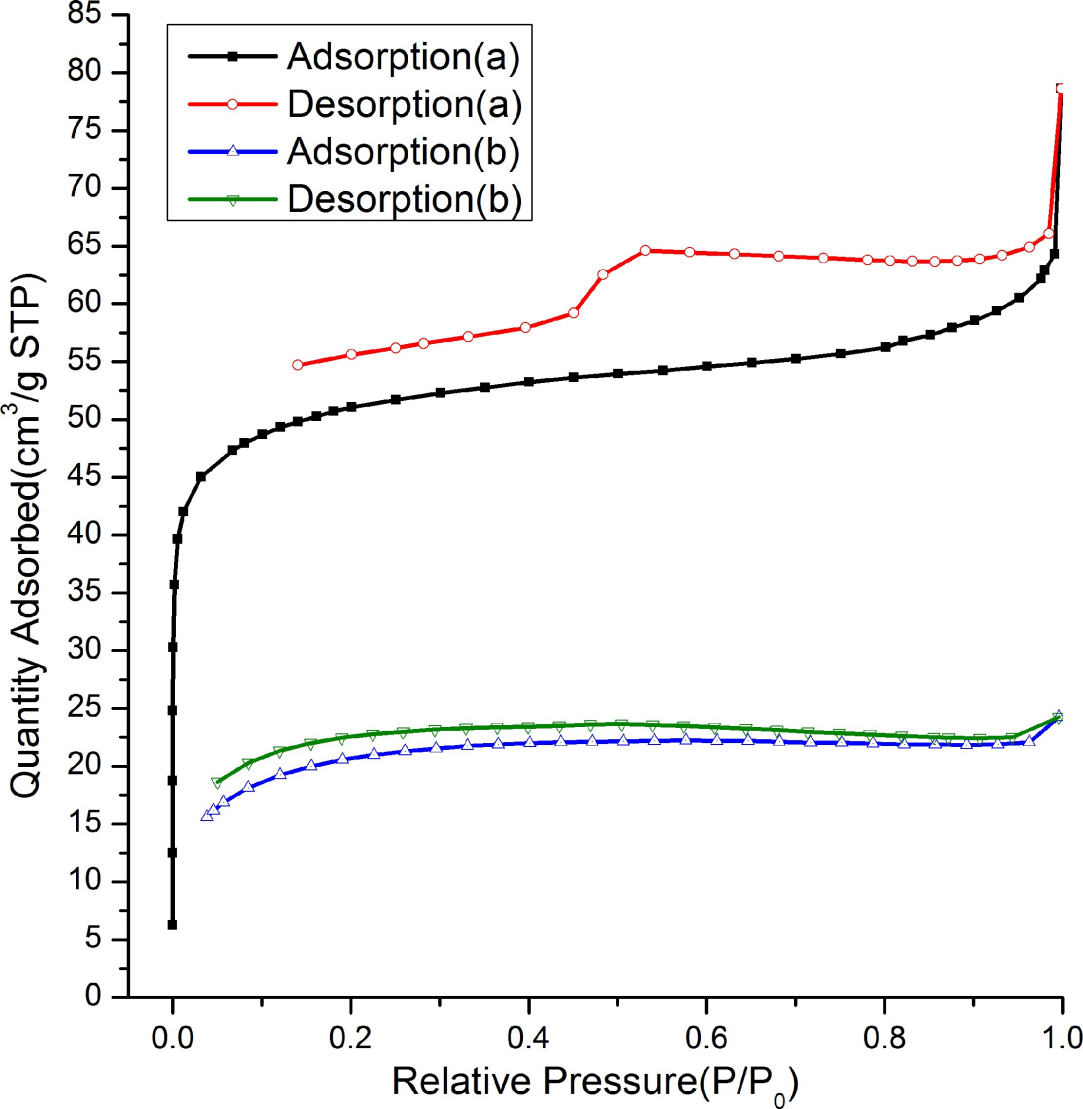
N_2_adsorption–desorption isotherms of (**a**) ethanol–HClextracted and (**b**)calcinedsilicas with the following condition: nTEOS:nCTAB:nDHAA:nNaOH:nH_2_O = 1:0.114:0.00457:0.5:60.

**Fig 7:**
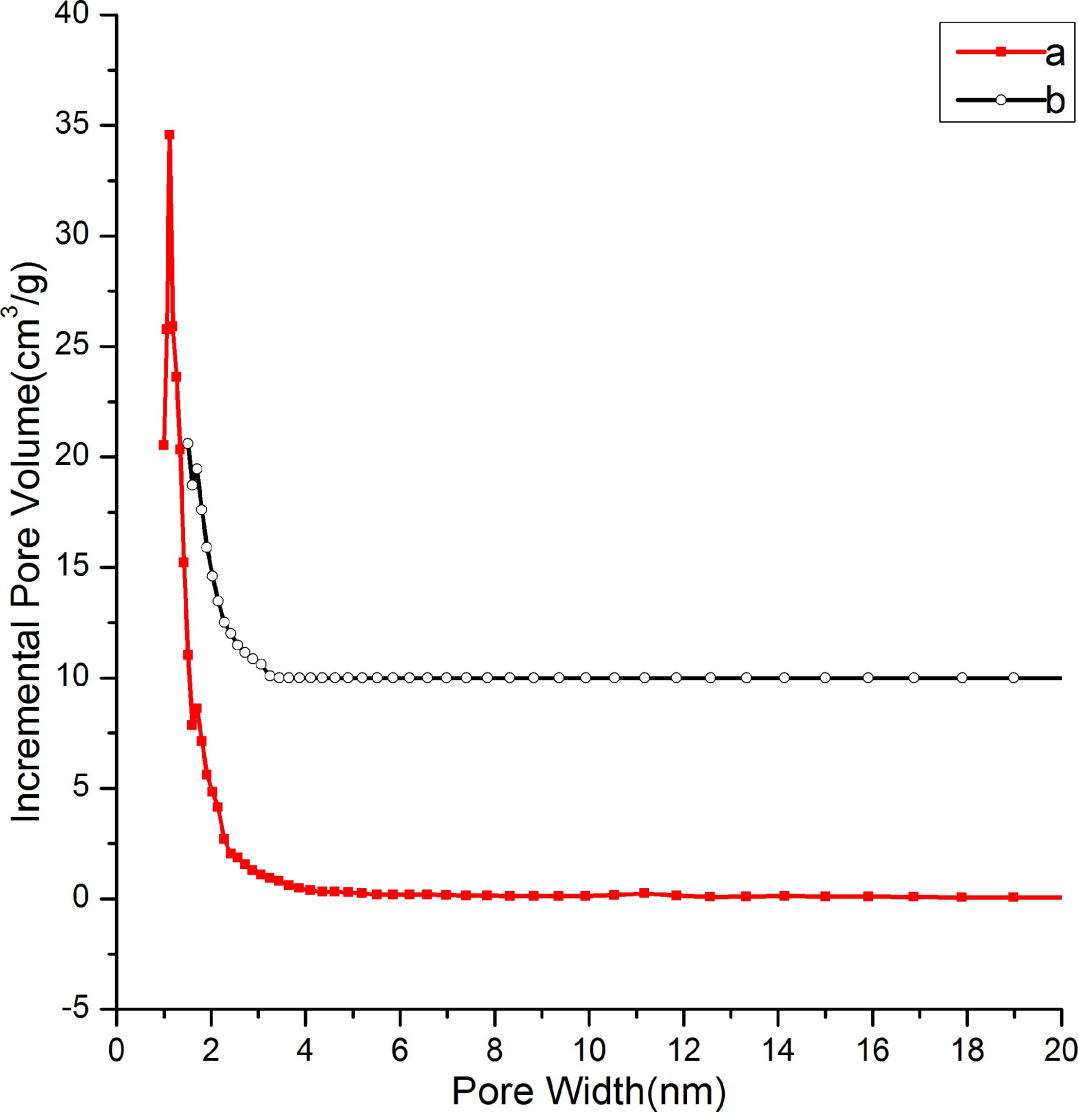
Pore size distributions of (**a**) ethanol–HClextracted and (**b**) calcinedsilicas with the following condition: nTEOS:nCTAB:nDHAA:nNaOH:nH_2_O = 1:0.114:0.00457:0.5:60.

Fig 6 plots the N_2_ adsorption–desorption isotherms of the ethanol–HCl extracted and calcined samples prepared with a mole ratio of nTEOS:nCTAB:nDHAA:nNaOH:nH_2_O = 1:0.114:0.00457:0.5:60.The ethanol–HCl extracted sample (Fig 6A) showed a transitional adsorption isotherm of typical I and IV adsorption behavior together with an H4 type hysteresis loop, suggesting the formation of a surpermicroporous structure. The desorption branch could not be closed with the adsorption branch, which may have been caused by interference of the templating agent that was not completely removed by the ethanol–HCl extraction. The calcined sample exhibited a II type adsorption isotherm (Fig 6B), and the value of quantity adsorbed was low, revealing that the sample was not rich in porosity after calcination. Fig 7 shows the DFT pore size distributions of the ethanol–HCl extracted and calcined samples prepared with a mole ratio of nTEOS:nCTAB:nDHAA:nNaOH:nH_2_O = 1:0.114:0.00457:0.5:60. A pore size distribution centered at about 1.12 nm could be detected for the ethanol–HCl extracted sample (Fig 7A). The surface area was169.34m^2^/g and the pore volume was0.12cm^3^/g. As for the calcined sample, a pore size distribution around 1.70 nm was observed (Fig 7B). The surface area was73.98 m^2^/g and the pore volume was0.03 cm^3^/g.

## Conclusions

Ordered supermicroporous silicas were synthesized by using a mixture of neutral primary amine dehydroabietylamine and long-chain cetyltrimethylammonium bromide as the templating agents. The dosages of DHAA, CTAB, and NaOH were the key factors for the formation of the lamellar supermicroporous structure. Even if only a small amount of DHAA is added to the sol-gel system of CTAB, the structure of the samples can be changed from cubic phase to lamellar phase. This phenomenon may have been caused by the solubilization of DHAA in CTAB micelles, which change the effective surfactant ion pair packing parameter. The dosage of CTAB should be moderate; too high or too low will decay the ordering degree of the lamellar structure. Only a suitable concentration of NaOH is beneficial to the formation of lamellar phase. A further increase inNaOH concentration will result in an ethanol-rich solvent, in which the DHAA will not solubilize in CTAB micelles, but adsorb at the hydrophilic headgroup–solvent interface. Accordingly, a transformation from lamellar phase to hexagonal phase occurred. When the molar ratio of components was nTEOS:nCTAB:nDHAA:nNaOH:nH_2_O = 1:0.114:0.00457:0.5:60, the lamellar sample possessed the highest ordering degree and the pore size was about 2 nm.

## Supporting information

S1 File Molecular structure of dehydroabietylamine. (PDF)
