# Supplementary figures and images for: Synthesis of ordered lamellar supermicroporous silica with rigid neutral and long-chain cationic composite templating route
Source: PLoS One. 2019 Apr 26;14(4):e0216117. doi: 10.1371/journal.pone.0216117 (PMC6485778; doi:10.1371/journal.pone.0216117)

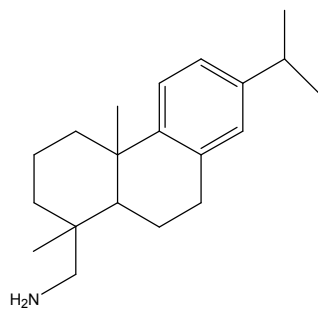

**S1 File.** Molecular structure of dehydroabietylamine

Supplement: S1 File — (PDF) [file pone.0216117.s001.pdf]
